# Superficial Heating Evaluation by Thermographic Imaging before and after Tecar Therapy in Six Dogs Submitted to a Rehabilitation Protocol: A Pilot Study

**DOI:** 10.3390/ani11020249

**Published:** 2021-01-20

**Authors:** Simona Valentini, Enrico Bruno, Caterina Nanni, Vincenzo Musella, Michela Antonucci, Giuseppe Spinella

**Affiliations:** 1 Department of Veterinary Medical Sciences, University of Bologna, Ozzano dell’Emilia, 40064 Bologna, Italy; giuseppe.spinella@unibo.it; 2 Veterinary Hospital “I Portoni Rossi”, Anzola dell’Emilia, 40011 Bologna, Italy; Enrico.Bruno@portonirossi.it (E.B.); caterina.nanni2@studio.unibo.it (C.N.); Michela.Antonucci@portonirossi.it (M.A.); 3 Department of Health Sciences, University Magna Graecia, 88100 Catanzaro, Italy; musella@unicz.it

**Keywords:** thermography, Tecar, superficial temperature, rehabilitation, dog

## Abstract

**Simple Summary:**

Tecar (energy transfer capacitive and resistive) is commonly used in dog rehabilitation as it can accelerate the healing process. Few studies have aimed to understand its effects on superficial tissues of the treated anatomic region. The aim of this study was to monitor by thermal camera the heating effects induced by Tecar on the surface of the region of application. The investigation was conducted on six dogs referred for Tecar therapy to treat muscle contractures (three dogs) or osteoarthritis (three dogs). Thermographic images and relative measurements were obtained by each region immediately before (T0), at conclusion (T1), and sixty seconds after the Tecar application (T2). Statistically significant differences were detected for mean temperature between T0 (32.42 ± 1.57 °C) and T1 (33.36 ± 1.17 °C) (*p* = 0.040) and between T1 and T2 (32.83 ± 1.31 °C) (*p* = 0.031).

**Abstract:**

Thermography is a non-invasive diagnostic method commonly used to monitor changes of the body surface temperature potentially induced by different conditions such as fever, inflammation, trauma, or changes of tissue perfusion. Capacitive-resistive diathermy therapy (such as energy transfer capacitive and resistive—Tecar) is commonly used in rehabilitation due to its diathemic effect secondary to blood circulation increase that could accelerate the healing process. The aim of this study was to monitor by thermal camera the diathermic effects induced by Tecar on the surface of the region of application. The investigation was conducted on six dogs referred for Tecar therapy to treat muscle contractures (three dogs) or osteoarthritis (three dogs). Eleven anatomical treated regions were recorded. Thermographic images and relative measurements were obtained by each region immediately before (T0), at conclusion (T1), and sixty seconds after the Tecar application (T2). Data were recorded and statistically analyzed. A comparison of temperature differences (maximum, minimum and mean values) between T0 and T1, T0 and T2, and T1 and T2 was performed by ANOVA test with Bonferroni post hoc (*p* ≤ 0.05). Statistically significant differences were detected for mean temperature between T0 (32.42 ± 1.57 °C) and T1 (33.36 ± 1.17 °C) (*p* = 0.040) and between T1 and T2 (32.83 ± 1.31 °C) (*p* = 0.031). Furthermore, there was no significant difference between the mean temperature at T0 and T2, demonstrating that superficial diathermic effect exhausted within 60 s.

## 1. Introduction

Thermography is an imaging technique that provides a quantification of superficial temperature using a color scale. In the medical field, thermography is considered a non-invasive diagnostic imaging technique that allows the detection of changes in body surface temperature [1]. Common advantages are the lack of direct contact and invasiveness, the absence of contamination, and high versatility [1,2]. The main disadvantage is that it does not show any ability to recognize the etiology of the disease, but only the thermal alteration [1]. Current applications in veterinary medicine include neoplasia diagnosis [3], assessment of orthopedic and neurological diseases [4,5,6,7,8,9,10,11,12] as well as the effects of exercise on thermal images [13,14,15]. Indeed, internal and surface temperatures are influenced by physical exercise [14]. In Greyhounds, the superficial temperatures measured from the gastrocnemius muscle are significantly higher after the race than at baseline [13] and Repac et al. demonstrated a significant increase in surface temperature of the biceps femoris and gracilis muscles before and after a 6-min walk in healthy dogs [15]. In contrast, eye temperature is unaffected by the length, moisture content and colour of hair-coat: it increases in response to physical exercise and could be used to assess arousal and frustration on performance in racing Greyhounds [16]. Moreover, thermography has been recently used to monitor behaviours of wild animals, as it can work during the night and remotely on moving animals [3]. In medicine, thermography allows the evaluation of changes in blood circulation induced by inflammatory or degenerative diseases, providing indications on site and evolution of the pathological processes, but it is unable to detect the etiology of the disease [1,3]. The main limitation is that it is affected by environmental factors (temperature, humidity, wind, etc.) as well as by intrinsic factors related to the animal (coat, size, circadian rhythms) [17].

Energy transfer capacitive and resistive therapy (Tecar) is a non-pharmacological diathermic modality that induces a tissutal heating increase. Its capability to increase blood flow is commonly considered to be the primary mode of improving circulation, promoting tissue healing, alleviating muscle and joint pain, and increasing connective tissue elasticity [18].

For these reasons Tecar therapy is used in healing processes and to treat specific disorders, such as pathologies of the joint capsule, arthritic process, muscle spasm and contracture, neuralgia, and oedema [19,20]. The main contraindications include suppurative processes, vascular diseases, neoplasia, and pregnancy [21]. It is also not recommended for direct application to the eyes, testicles, and growth plate cartilage [21]. In overweight animals, particular attention must be paid to avoid overheating of the poorly vascularized adipose tissue, which does not allow adequate vasodilation and, consequently, a rapid and effective cooling [22,23].

Despite some preliminary evidences about its clinical efficacy, knowledge of the physiologic responses induced in animals by Tecar therapy is limited and, unlike other methods such as warm compresses, to date, the effects of the heating induced by Tecar therapy on canine superficial tissues have not been described.

Therefore, the aim of this clinical study was to evaluate, by thermo camera, the superficial heating and relative short-term thermal changes in dogs submitted to Tecar therapy for pathologies related to the musculoskeletal system (three dogs with muscle contracture and three with osteoarthritis).

## 2. Materials and Methods

All procedures described in the manuscript were performed in routine clinical activity by licensed veterinarians following a normal standard protocol approved by the owners of the dogs (informed consent). Moreover, this study was carried out in accordance with the relevant guidelines and regulations required by Italian Veterinary Clinical Practice (as reported in FNOVI-Federazione Nazionale Ordini Veterinari Italiani-Deontological Guidelines, art. 15).

A portable thermo camera, AVIO TVS-200 EX (Inprotec IRL srl, Milano, Italy), long wave (8–14 µm), equipped with a microbolometer detector and resolution of 320 × 240 pixels and set on a temperature range between 20 and 39 °C, was used. The field of view was 30.6° × 23.1°.

Thermography was performed on six dogs referred to the Physiotherapy Unit “Kinetic” of the Veterinary Hospital “I Portoni Rossi” (Italy) for Tecar therapy (as part of a rehabilitation protocol) to treat muscle contracture (only 3 dogs) or osteoarthritis (only 3 dogs). All dogs had similar coat colour. As three dogs needed Tecar therapy application in different regions, eleven final regions of interest (ROIs) were investigated.

For each dog, breed, age, gender, weight, and reason for treatment were recorded. The investigation was conducted in a room with artificial light and ambient temperature controlled at 26 °C by a centralized control system. Previously, the dogs had been kept in a room with similar environmental characteristics for at least 2 h. The patients were accustomed to the absence of the owner as they were submitted to daily in-clinic treatments in order to reduce stress and avoid vasomotor responses that could alter our examination and relative outcomes. Afterwards, in order to minimize environmental artifacts, dogs were positioned on the floor or on a non-metallic platform and the manipulations by the operators were reduced to a minimum necessary for patient positioning.

Tecar therapy was performed with a d-VET 900 Pet Diatherm System (White Medical & Beauty, Bologna, Italy), with 1 MHz frequency of work and 750 W maximum input power and 100 W maximum output power. Medium power level protocols (35–40% of maximum out power) were set for the medical treatments. The operators (DVM, Certified Canine Rehabilitation Practitioner) avoided any physical contact with the animal; the duration of a single treatment was 15 min.

**Figure 1:**
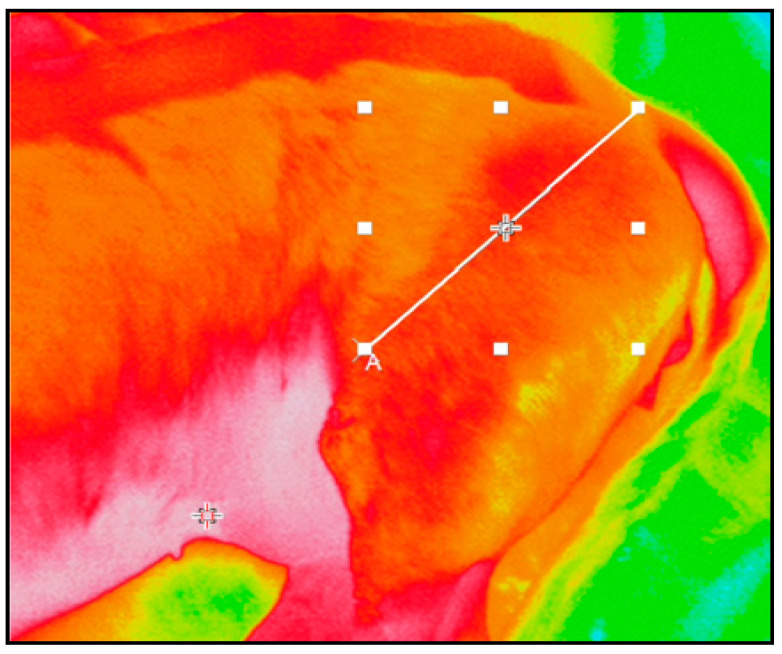
Dachshund, female, 5 yo. Left thigh. In the image squared shaped region (area) of interest (ROI) and diagonal (line) are defined. “A” defines the origin of the diagonal.

Images were obtained by each anatomical region immediately before (T0), at the end (T1), and 60 s after the end of Tecar therapy (T2). The distance between the dog and the camera was 40 cm: the procedure did not entail any constrictive or invasive maneuver that could affect the animal’s well-being. The images were acquired by a camera display. Data (images) were recorded and analyzed with Inf Rec Analyzer NS9500 Lite software (Nippon Avionics Co.LTD, Yokohama-Shi, Japan). Squared or rectangular shaped regions (areas) of interest (ROIs) were defined within each image. Areas were selected on the basis of the area of application of the Tecar therapy probe following the clinical evaluation and the diagnostic imaging. For each area, maximum, minimum, and mean temperatures were extrapolated at T0, T1, and T2. The same data were further obtained by the diagonal (line) of each single area (Figure 1). Profile and thermal diagrams were also recorded.

Data were submitted to descriptive (mean ± standard deviation and median) and analytic statistical analysis in order to investigate any significant variation in the maximum, minimum, and mean temperatures of both areas and lines in T0, T1, and T2. Comparison of temperature differences (maximum, minimum, and mean values) between T0 and T1, T0 and T2, and T1 and T2 within the areas and the lines (diagonals) were performed by use of ANOVA with a Bonferroni post hoc test. The significance level was set for *p*-values ≤ 0.05. Statistical analyses were performed using dedicated software: Stata version 15 (StataCorp, College Station, TX, USA–2017).

## 3. Results

Six client-owned dogs (three males and three females) of different breeds (two Dachshunds, two mixed-breed dogs, one Boxer, and one Dobermann), mean age of 7.3 ± 3.59 years (median age 7 years; range 3–13 years) and mean weight of 19.5 ± 15.9 kg (median weight 10.5; range 7–50 kg) were included in the study. Three dogs were short-haired and three were curly-haired with reduced undercoat. Tecar therapy was applied in three dogs affected by muscle contractures and in three dogs for osteoarthritis. The final ROIs analyzed by thermography were 11 (Table 1).

Thermal color analysis: Skin temperature following Tecar therapy (T1) increased, as manifested by warmer intense red color areas, turning toward progressively lighter shades (pink–white). After 60 s (T2), colors returned to the initial pattern. The increase in superficial temperature was also confirmed by the thermal diagram and thermal profile, with temperatures measured on the line in T1 being much higher than those in T0. Within our short-haired patients, the Doberman (dog n° 6) showed a basal temperature slightly higher (about 1 °C) than the other dogs, with no apparently pathological reason. A higher temperature value in T0 probably justified the lack of thermal changes at T1 in this dog. Generally, images at T1 showed a warmer thermal pattern compared to T0, with a color change toward bright red, that returned to a color pattern similar to that at T0 within 60 s (T2). The thermal profiles and thermal diagrams also confirmed this assessment.

Descriptive analysis: Areas showed a mean surface thermal increase at T1 (0.94 ± 0.99 °C) and a rapid return to the initial values (<60 s). The comparison between mean values at T0 in curly-haired (n° 2, 3, 4) and short-haired (n° 1, 5, 6) dogs showed a thermal difference of about 2 °C, as the mean value at T0 in curly-haired dogs was 31.55 ± 0.88 °C, while in short-haired dogs it was 33.49 ± 1.40 °C. The thermal increase (T0–T1) of mean temperature in curly-haired dogs was 1.03 ± 0.34 °C, while in short-haired dogs it was 0.83 ± 1.41 °C.

In areas, the mean of maximum temperature values and standard deviation (SD) at T0 was 35.32 ± 1.10 °C (median 35.72 °C); at T1, 36.17 ± 0.99 °C (median 36.08 °C), and at T2, 35.53 ± 1.21 °C (median 35.81 °C). The mean of minimum temperature values and SD at T0 was 29.59 ± 2.23 °C, at T1, 30.00 ± 2.11 °C, and at T2, 29.76 ± 1.70 °C; with median values of 29.8 °C, 28.45 °C, and 29.12 °C, respectively. The mean of the mean temperature values and SD was 32.42 ± 1.57 °C at T0, 33.36 ± 1.17 °C at T1, and 32.83 ± 1.31 °C at T2. The median of the mean temperatures calculated at T0, T1, and T2 were 32.07 °C, 33.58 °C, and 32.81 °C, respectively. All values, rounded to the decimal, are summarized in Table 2.

In relation to the lines (diagonal of ROI), the mean of maximum temperature values and SD at T0, T1, and T2 were 34.52 ± 1.07 °C, 35.50 ± 0.52 °C, and 34.57 ± 1.43 °C, respectively, with related median values at T0, T1, and T2 of 34.82 °C, 35.36 °C, and 34.73 °C. The mean of minimum temperature values and SD at T0, T1, and T2 were 30.41 ± 2.39 °C, 31.29 ± 1.72 °C, and 30.84 ± 1.99 °C; the medians were 29.8 °C, 30.75 °C, and 30.08 °C, respectively. The mean of the mean temperature values was 32.46 ± 1.61 °C at T0, 33.54 ± 1.14 °C at T1, and 32.89 ± 1.57 °C at T2 and the median values were 32.15 °C, 33.75 °C, and 33.43 °C, respectively. All values, rounded to the decimal, are summarized in Table 2.

**Figure 2:**
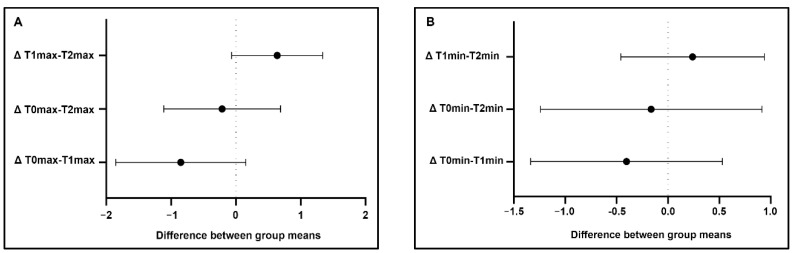

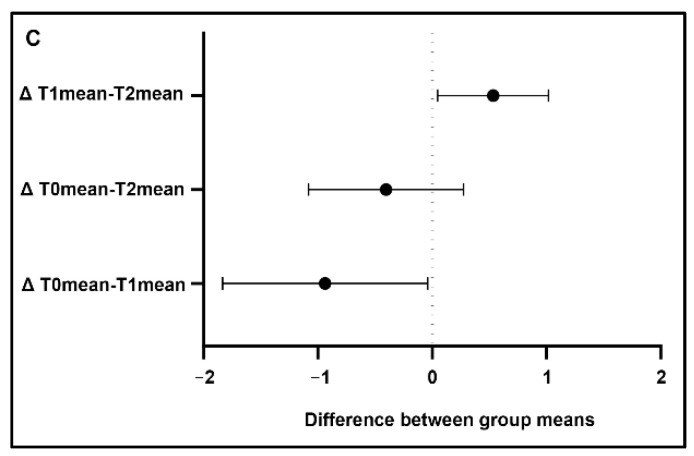
Maximum (**A**), minimum (**B**) and mean (**C**) temperature differences in the areas: the intersection with the dotted line identifies “not statistical significance”. The mean temperature differences T0–T1 and T1–T2 are significant (**C**). Δ = temperature difference in °C.

**Figure 3:**
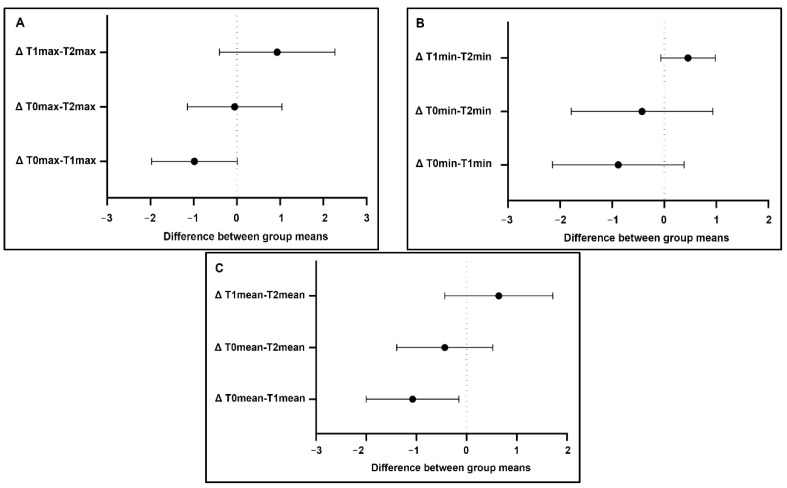
Maximum (**A**), minimum (**B**) and mean (**C**) temperature differences in the lines (diagonal): the intersection with the dotted line identifies “not statistical significance”. Only the mean difference in temperature T0–T1 is significant (**C**). Δ = temperature difference in °C.

Statistical analysis: The statistical analysis did not reveal significant differences in the maximum and minimum temperature values between T0 and T1, T0 and T2, and T1 and T2 either in the areas and lines (*p* > 0.05). Additionally, analysis of the maximum and minimum temperatures in the areas of interest showed a similar effect of Tecar therapy, with a mild increase in temperature between T0 and T1 and a decrease between T1 and T2. Statistically significant differences in the mean temperature were observed between T0 and T1 (*p* = 0.022) in the lines, and between T0 and T1 (*p* = 0.040) and T1 and T2 (P = 0.031) during area evaluations (Figure 2 and Figure 3). The clinical cases with no increase at T1 were generally associated with a higher basal value at T0. A containment of the thermal rise within physiological limits was therefore detected. 

## 4. Discussion

In veterinary medicine, there are few articles on the effects of Tecar therapy in dogs [19,20,21]. In particular, as far as the authors are aware, there are no indications regarding the heating of the superficial areas induced on the treated region. A pilot study by Clijsen et al. found that the resistive mode can induce statistically significant changes in the skin temperature of healthy human volunteers [24].

Our results showed basal temperatures (T0) different than those detected in intact coat Labrador Retrievers by Loughin and Marino (2007), who examined dogs in ambient temperature (21 °C), lower than that reported in our investigation (26 °C) [4]. Environmental conditions and coat length could probably account for these different findings. However, Kwon and Brundage (2019) examined short-coated and curly-coated dogs and reported a mean superficial body temperature greater than 29 °C, similar to our findings [25]. A difference in mean temperature value at T0 was found in our outcomes between curly-haired and short-haired dogs, but this difference was in agreement with the literature [25].

In our cases, the increase of mean temperature from T0 to T1 was about 1 °C despite the high variability between the different dogs (mean and SD = 0.94 ± 0.99 °C): that slight increase should confirm the contained and safe effect of Tecar therapy on the surface of the treated region.

Furthermore, there was no significant difference between the mean temperatures at T0 and T2 in the areas, demonstrating that skin cooling restores the basic values within 60 s. These results confirm the limited superficial thermal increase caused by Tecar therapy, but also the short duration of its effect, validating the moderate surface involvement and the high degree of safety of Tecar therapy.

When the lines were analyzed, the only statistically significant data were observed between T0 and T1. However, it should be considered that the lines examined a limited distribution of the points and the relative temperatures could not perfectly correspond to the range of treated anatomic areas. For this reason, it is our opinion that the analysis of the areas could assume a greater significance than the lines, because a wide number of pixels were analyzed.

Unfortunately, the scientific literature does not report measurements of superficial temperature and the persistence of the diathermic effect for all diathermy methods. Some indications were reported for therapeutic ultrasound (TUS) and hot compresses. Levine et al. (2001) reported a 3 °C and 4.6 °C temperature rise at a 1.0 cm depth after TUS application at an intensity of 1.0 W/cm^2^ and 1.5 W/cm^2^, respectively. Tissue temperature returned to baseline value within 10 min after a 10-min treatment [26]. Although the evaluation was not made on the skin surface but at 1 cm of depth, it seems likely that skin heating could be much more marked than what occurred during Tecar-therapy, which consequently should be preferable to TUS during inflammatory or traumatic skin pathologies. Millard et al. (2013) declared that the mean increase of maximum temperature induced by warm compresses at 0.5 cm of depth was 4.14 °C and 4.56 °C after 10 and 20 min of heat application, respectively. In their investigation, the return to baseline occurred not before 40 min [22]. Thus, although the warm compress is practical and simple to use, in specific situations (such as acute dermatopathies), this technique could prove excessively traumatizing for the tissues compared to Tecar therapy, as the thermal increase is not only higher, but also longer lasting.

Some technical aspects of this preliminary investigation deserve careful consideration. Ambient temperature and humidity measurements are critical factors in the thermographic examination: for this reason, temperature was recorded and maintained stable during the entire phase of image acquisition. Conversely, environmental humidity was not monitored. However, the thermal camera was set up for a conditioned indoor environment and no “outdoor” activity was planned. Moreover, a previous publication showed that changes in the range of humidity (0 to 100%) entailed limited effects on thermography if room temperature was constant and the distance between ROI and the camera was not greater than 1 m, as was scheduled in our study [27]. Surfaces, on which patients rest, could also affect outcomes. These surfaces should be standardized and not made of metallic material to minimize artifacts. For this reason, steel tables or containment cages were avoided. As previously discussed, the coat has also been implicated as a cause of different interpretations of thermographic images [1,3,4,17,25]. In our experience, we did not find any difficulty during the interpretation and analysis of images for curly-haired dogs with reduced coat, but we did not examine long-haired dogs.

This study has some limitations. A limitation is the lack of a control group, but although thermography is a non-invasive diagnostic imaging method, healthy dogs were not included for ethical reasons. Moreover, enrolled dogs were different for age, breed, coat length, size, and their temperatures could show physiological differences that could influence the standardization of whole thermal analysis. The length of the coat could influence the measurement of the skin temperature, but clinical application of Tecar therapy does not require shaving the treated area. In order to avoid any misinterpretation, for skin temperature evaluation an infra-red thermometer could be used alongside thermography, as described in a recent study of Hepps Keeney and collaborators [28]. Although data obtained showed that the basal temperatures were similar to those reported in the literature, it will be necessary in the future to extend the study to a greater number of patients in order to validate these preliminary outcomes.

## 5. Conclusions

Thermography appears to be a rapid, relatively easy, and effective diagnostic technique for thermal analysis. Moreover, it allows evaluation of the skin temperature without any contact with the patient, thereby minimizing stress for the animal. Furthermore, this technique demonstrates that the superficial temperature is not particularly influenced by diathermic effects of Tecar therapy, reporting only mild and short-term thermal increases and thus proving the safety of the Tecar therapy use at reported power level protocols. It is the authors’ opinion that further investigations are required to confirm this preliminary investigation on this specific, highly widespread diathermic modality.

## Figures and Tables

**Table 1:** Signalment of dogs underwent to thermographic investigation, anatomical regions treated by Tecar theraphy, disease treated by Tecar therapy (DTT). CM = Castrated Male, F = Female, M = Male, SF = Spayed Female. RT = right thigh; LT = left thigh: RF = right forelimb; LF = left forelimb.

N°	Breed	Sex	Age (Year)	Weight (Kg)	Anatomical Region	DTT
1	Boxer	CM	3	31	RT	Muscle contracture
2	Dachshund	F	5	8	LT	Muscle contracture
3	Mixed-breed	M	13	12	RT, LT, RF, LF	Muscle contracture
4	Mixed-breed	F	4	9	C2–C5	Cervical spondylarthrosis
5	Dachshund	SF	9	7	RT, LT	Bilateral hip arthrosis
6	Dobermann	CM	10	50	RT, LT	Bilateral stifle arthrosis

**Table 2:** Mean ± SD and Median and Interquartile Range (IQR) of maximum, minimum and mean temperature values in the areas and the lines at T0, T1 and T2. All values are expressed in °C.

	AREA	AREA	AREA	LINE	LINE	LINE
	Tmax	Tmin	Tmean	Tmax	Tmin	Tmean
Mean ± SD						
T0	35.3 ± 1.1	29.6 ± 2.2	32.4 ± 1.6	34.5 ± 1.1	30.4 ± 2.4	32.4 ± 1.6
T1	36.2 ± 1.0	30.0 ± 2.1	33.4 ± 1.2	35.5 ± 0.5	31.3 ± 1.7	33.5 ± 1.2
T2	35.5 ± 1.2	29.8 ± 1.7	32.8 ± 1.3	34.6 ± 1.4	30.8 ± 2.0	32.9 ± 1.6
Median (IQR)						
T0	35.7 (34.6–35.9)	29.8 (27.3–31.1)	32.1 (31.3–33.9)	34.8 (33.4–35.5)	29.8 (28.6–32.3)	32.1 (31.0–33.9)
T1	36.1 (35.4–37.1)	28.4 (28.3–32.4)	33.6 (32.9–34.4)	35.4 (35.1–36.1)	30.7 (30.2–33.0)	33.7 (32.9–34.3)
T2	35.8 (34.8–36.5)	29.1 (28.4–31.4)	32.8 (31.6–33.7)	34.7 (33.5–35.6)	30.1 (29.1–33.2)	33.4 (31.1–33.9)

## Data Availability

All data is contained within this article. Interested qualified researchers may request further information by contacting the corresponding author.
